# Pharmacokinetics, safety, and dose optimization of GST-HG171 in renal impairment: insights from physiologically based pharmacokinetic modeling

**DOI:** 10.1128/aac.01541-25

**Published:** 2026-07-15

**Authors:** Qingmei Li, Min Wu, John Mao, Tianxiang Zhang, Wenhao Yan, Xiuping Yan, Chuanjing Li, George Zhang, Yanhua Ding, Jiajia Mai, Hong Zhang

**Affiliations:** 1 Department of Pediatric Nephrology, Children’s Medical Center, The First Hospital of Jilin University, Jilin, China; 2 Phase I Clinical Research Center, The First Hospital of Jilin University, Jilin, China; 3 Fujian Akeylink Biotechnology Co., Ltd., Fuzhou, Fujian, China; Chinese Academy of Medical Sciences & Peking Union Medical College, Beijing, China

**Keywords:** clinical trial, renal impairment, pharmacokinetics, safety, GST-HG171, COVID-19, PBPK

## Abstract

GST-HG171 is an orally administered inhibitor of the 3C-like protease that has been approved in China for the treatment of mild to moderate COVID-19. When co-administered with ritonavir, GST-HG171 is eliminated by the kidney. Therefore, this study evaluated the impact of renal impairment on its pharmacokinetics (PK), safety, and tolerability. A total of 24 participants were enrolled: 8 with normal renal function, 8 with mild renal impairment, and 8 with moderate renal impairment. Participants received 100 mg ritonavir at −12, 0, 12, and 24 h, with a single 150 mg dose of GST-HG171 co-administered at 0 h to maintain CYP3A inhibition. Compared with healthy subjects, mild renal impairment increased GST-HG171 exposure; the geometric least-squares mean ratios (90% confidence intervals) for *C*_max_, AUC_0*–t*_, and AUC_0–∞_ were 118.15 (99.38–140.46), 139.48 (111.21–174.95), and 138.46 (111.19–172.42), respectively. Moderate impairment further increased exposure, with ratios of 131.81 (108.34–160.36), 186.18 (150.47–230.36), and 182.64 (148.03–225.33), respectively. Renal clearance was reduced by 22.7% and 52% in the mild and moderate impairment groups, respectively. GST-HG171 was generally tolerated across all groups, but adverse reactions related to hypertriglyceridemia should be monitored. Physiologically based PK modeling supported dose reductions for patients with renal impairment, recommending GST-HG171/ritonavir 100/100 mg twice daily for mild impairment and 50/100 mg twice daily for moderate impairment, administered for 5 days.

**CLINICAL TRIALS:**

This study is registered with ClinicalTrials.gov as NCT06106126.

## INTRODUCTION

As of 21 July 2024, a total of 775 million confirmed cases and 7 million deaths have been reported globally since the onset of the pandemic (1). Although COVID-19 has transitioned from a pandemic to an endemic disease, it continues to cause considerable morbidity and mortality, particularly in regions where vaccination coverage remains low. Current clinical recommendations support the use of oral antiviral medications for nonhospitalized individuals with mild or moderate COVID-19 who are at increased risk of developing severe disease (2–4). Among these, 3C-like (3CL) protease inhibitors have drawn significant attention. In clinical trials, nirmatrelvir co-administered with ritonavir reduced the risk of progression to severe disease by 89% compared with placebo, with no major safety concerns reported (5–9).

Although nirmatrelvir has been approved for clinical use in the treatment of COVID-19, further expanding the antiviral therapy repertoire and providing patients with evidence-based, diversified treatment options may improve overall outcomes and patient benefits. GST-HG171 is a novel, potent, and selective oral 3CL protease inhibitor. Preclinical studies identified cytochrome P450 (CYP) enzymes, particularly CYP3A, as primarily responsible for the metabolism of GST-HG171. In a first-in-human clinical study, including single-ascending-dose, multiple-ascending-dose, and drug–drug interaction cohorts, ritonavir (a strong CYP3A4 inhibitor) increased GST-HG171 exposure (*C*_max_ and area under the concentration–time curve [AUC]) in healthy subjects by three- to six-fold, thereby ensuring trough concentrations well above the *in vitro* EC_50_ (10). A subsequent pharmacokinetic (PK) study in subjects with hepatic impairment showed that mild to moderate hepatic dysfunction had minimal impact on drug exposure, indicating that dose adjustment is not required in this population (11). Based on these findings, a regimen of 150 mg GST-HG171 with 100 mg ritonavir was selected for Phase II/III trials, in which GST-HG171 shortened the median time to symptom recovery and accelerated viral clearance compared with placebo (12). GST-HG171 demonstrated superior antiviral activity compared with nirmatrelvir in preclinical studies both *in vitro* and *in vivo*. In standard-risk patients with mild-to-moderate COVID-19, nirmatrelvir/ritonavir showed no significant effect on symptom resolution; GST-HG171, by contrast, supports recovery across all risk levels (12).

The medication has received approval from China’s National Medical Products Administration for the treatment of mild to moderate COVID-19 in China. Chronic kidney disease (CKD) can alter drug metabolism by reducing hepatic clearance mediated by cytochrome P450 enzymes and other pathways (13). Uremic toxins and oxidized protein products can suppress transcriptional activation, downregulate gene expression, and directly inhibit CYP3A4 and transporter activities (14, 15). In addition, patients eligible for GST-HG171 therapy may have renal impairment, which is a known risk factor for severe COVID-19 (16, 17). Because renal dysfunction may affect the PK profile of GST-HG171 and influence its safety, tolerability, and efficacy, PK and safety studies comparing subjects with renal impairment and healthy controls are needed. Here, we describe the pharmacokinetics, safety, and tolerability of a single oral dose of 150 mg GST-HG171 co-administered with 100 mg ritonavir in patients with mild or moderate renal impairment and compare these outcomes with those in healthy controls. Based on these findings, we also discuss dose adjustment recommendations.

## RESULTS

### Baseline characteristics of the subjects

We first assessed baseline characteristics. Of the 66 subjects screened, 24 met eligibility criteria and were enrolled, whereas 42 failed screening, primarily due to inclusion/exclusion criteria; of these, 38 were excluded and four served as backups. All 24 subjects (16 men and 8 women; ethnicity: 21 Han and 3 Manchu) completed the study according to protocol. The mean age was 48.63 ± 8.83 years, the mean weight was 70.35 ± 8.19 kg, and the mean body mass index (BMI) was 25.63 ± 2.26 kg/m². Baseline demographic characteristics were comparable across the normal renal function, mild impairment, and moderate impairment groups (Table 1).

**TABLE 1:** Demographics and baseline characteristics of participants by renal function group^*a*^

Baseline parameter	Mild renal impairment (*N* = 8)	Moderate renal impairment (*N* = 8)	Normal renal function (*N* = 8)
Age, years	44.13 ± 7.22	51.13 ± 12.29	50.63 ± 4.24
Sex (*N* [%])			
Male	6 (75.0)	5 (62.5)	5 (62.5)
Female	2 (25.0)	3 (37.5)	3 (37.5)
Ethnicity (*N* [%])			
Han	7 (87.5)	7 (87.5)	7 (87.5)
Weight, kg	72.64 ± 10.02	68.01 ± 8.95	70.41 ± 5.24
BMI, kg/m^2^	25.00 ± 2.20	26.13 ± 2.95	25.75 ± 1.58

^*a*^ Data are presented as mean ± SD.

### Pharmacokinetics of GST-HG171

**Fig 1:**
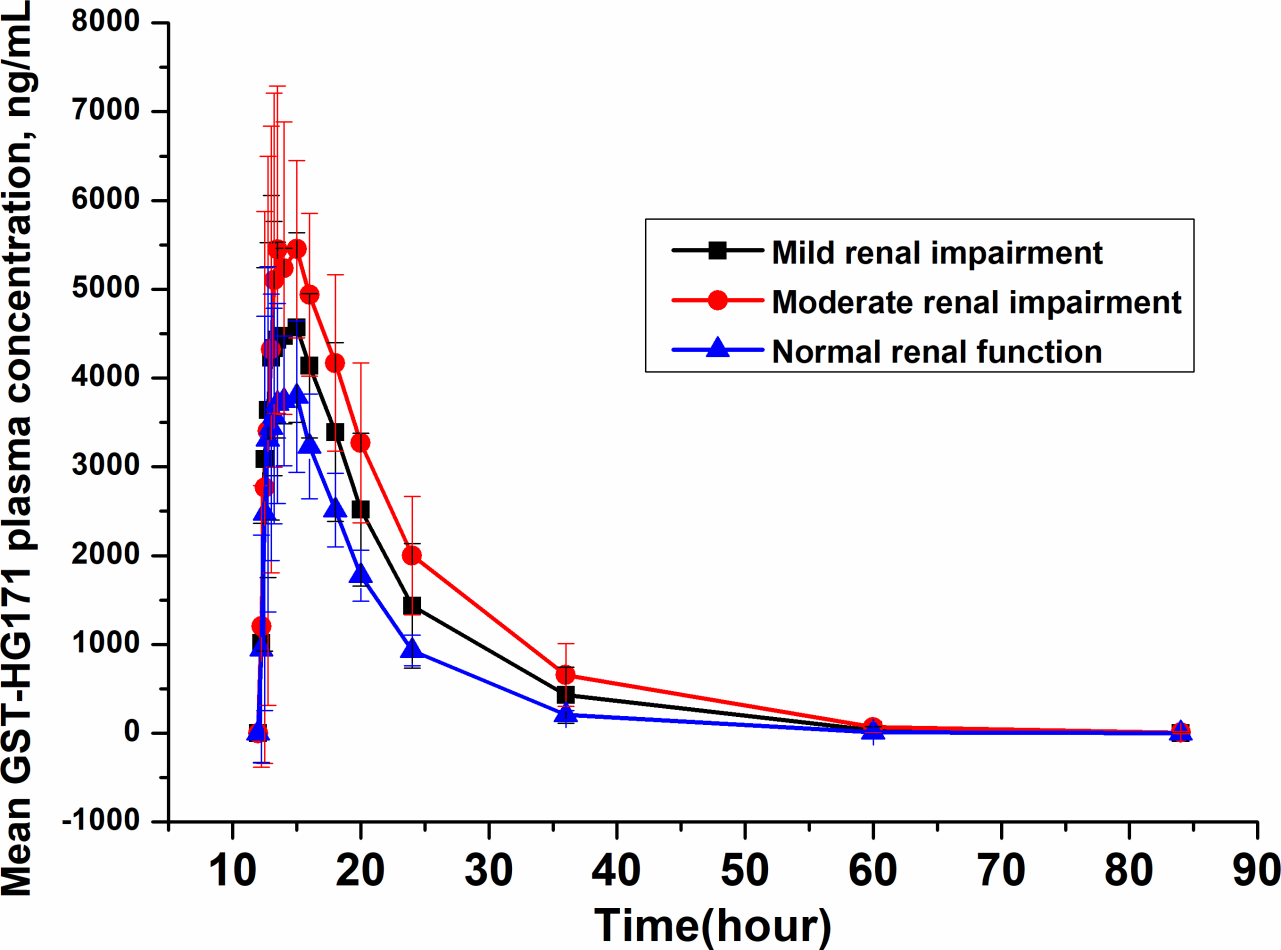
Mean plasma concentration–time profiles of GST-HG171 by renal function group. Plasma concentrations of GST-HG171 following a single 150 mg oral dose with ritonavir co-administration in subjects with normal renal function, mild renal impairment, and moderate renal impairment. Data are expressed as mean ± SD.

Subsequently, the pharmacokinetic behavior of GST-HG171 was evaluated following a single 150 mg oral dose administered with ritonavir. Plasma concentration–time curves were broadly similar in shape across groups (Fig. 1). Pharmacokinetic parameters are presented in Table 2. For GST-HG171, in subjects with mild renal impairment, the geometric mean ratios (mild vs normal) and corresponding 90% confidence intervals for *C*_max_, AUC_0*−t*_, and AUC_0−∞_ were 118.15 (99.38−140.46), 139.48 (111.21−174.95), and 138.46 (111.19−172.42), respectively (Table 3). When comparing subjects with moderate renal impairment to those with normal renal function, the corresponding geometric mean ratios (moderate vs normal) were 131.81 (108.34−160.36) for *C*_max_, 186.18 (150.47−230.36) for AUC_0–*t*_, and 182.64 (148.03−225.33) for AUC_0−∞_ (Table 3).

**TABLE 2:** Pharmacokinetic parameters of GST-HG171 following single-dose administration with ritonavir, stratified by renal function group

Parameter	Mild renal impairment (*N* = 8)	Moderate renal impairment (*N* = 8)	Normal renal function (*N* = 8)
AUC_0*–t*_ (h*ng/mL)	52,868.27 ± 17,897.27	70,136.13 ± 22,275.80	36,457.85 ± 5,027.09
AUC_0–∞_ (h*ng/mL)	53,663.18 ± 17,815.22	70,493.11 ± 22,286.71	37,326.59 ± 4,874.98
*C*_max_ (ng/mL)	5,536.39 ± 877.68	6,242.27 ± 1,445.18	4,719.84 ± 918.03
CL/F (L/h)	3.06 ± 0.97	2.31 ± 0.67	4.09 ± 0.63
*T*_max_^*a*^ (h)	1.13 (0.7506–3.0003)	1.75 (0.7508–3.0053)	1.25 (0.5003–3.0003)
*t*_1/2_ (h)	6.02 ± 1.40	6.91 ± 1.32	5.52 ± 1.14
AE_0–72h_ (mg)	66.5 ± 11.7	58.9 ± 7.3	67.1 ± 14.9
Fe_0–72h_ (%)	44.36 ± 7.84	39.28 ± 4.87	44.74 ± 9.98
CL_*r*_ (L/h)	1.43 ± 0.69	0.89 ± 0.19	1.85 ± 0.37

^*a*^ Median (min-max); data are presented as mean ± SD.

**TABLE 3:** Geometric mean ratios (90% CI) of *C*_max_ and AUC (test vs normal renal function) derived from ANOVA

Parameter	Geometric mean ratio (90% CI) (%)	
	Mild renal impairment vs normal renal function	Moderate renal impairment vs normal renal function
*C*_max_	118.15 (99.38–140.46)	131.81 (108.34–160.36)
AUC_0–*t*_	139.48 (111.21–174.95)	186.18 (150.47–230.36)
AUC_0–∞_	138.46 (111.19–172.42)	182.64 (148.03–225.33)

The time to maximum observed plasma concentration (*T*_max_) of GST-HG171 was similar across all groups, with median values ranging from 1.13 to 1.75 h. Relative to the group with normal renal function, CLr was reduced by approximately 22.7% in the mild impairment group and by 52.0% in the moderate impairment group. CL/F showed a similar reduction, and the elimination half-life was prolonged in proportion to the degree of renal impairment. The 72-h urinary excretion (adverse event [AE]_0–72h_) and cumulative excretion fraction (Fe_0–72h_) in the mild impairment group were similar to those in the normal group. In contrast, in the moderate impairment group, the values decreased to about 88% of those observed in the normal group.

### Safety and tolerability

We then evaluated safety and tolerability. No deaths, serious AEs, or study discontinuations occurred. A total of 8 of 24 subjects (33.3%) experienced treatment-emergent adverse events (TEAEs), of whom 7 (29.2%) had events considered related to GST-HG171. One subject (B001) experienced a grade III TEAE of hypertriglyceridemia, which was grade I at baseline. Another subject (B006) experienced a grade II TEAE of hypertriglyceridemia, which was also grade I at baseline. All remaining TEAEs were grade I in severity. All TEAEs resolved completely without sequelae, and none led to treatment discontinuation or study withdrawal. The incidence of adverse reactions was 12.5% (1/8) in both the normal and mild groups but markedly higher in the moderate impairment group (62.5%, 5/8). Hypertriglyceridemia was the most frequent adverse reaction in the moderate impairment group (25%). No clinically meaningful changes were detected in laboratory parameters, electrocardiogram (ECG), or vital signs (Table 4).

**TABLE 4:** Treatment-emergent adverse events (TEAEs) by renal function group

	Mild renal impairment (*N* = 8)		Moderate renal impairment (*N* = 8)		Normal renal function (*N* = 8)	
	Number of TEAEs	Percentage (*n*/*N*)	Number of TEAEs	Percentage (*n*/*N*)	Number of TEAEs	Percentage (*n*/*N*)
Increased blood uric acid	0	0	1	12.5% (1/8)	1	12.5% (1/8)
Increased blood glucose	0	0	1	12.5% (1/8)	0	0
Increased white blood cell count	0	0	1	12.5% (1/8)	0	0
Increased neutrophil count	0	0	1	12.5% (1/8)	0	0
Diarrhea	1	12.5% (1/8)	1	12.5% (1/8)	0	0
Vomiting	1	12.5% (1/8)	0	0	0	0
Hypertriglyceridemia	0	0	2	25.0% (2/8)	0	0
Hyperphosphatemia	0	0	1	12.5% (1/8)	0	0
Dizziness	1	12.5% (1/8)	0	0	0	0

### PBPK modeling and dose recommendations

Finally, we applied physiologically based pharmacokinetic (PBPK) modeling to guide dosing in patients with renal impairment. The model, developed using clinical data (single administration of GST-HG171 or a combination of GST-HG171 and ritonavir) from both healthy and renally impaired subjects (9), demonstrated good predictive accuracy, with geometric mean fold errors of 1.12 for AUC and 1.20 for *C*_max_ (Fig. S1 and Table S2). With increasing severity of renal impairment, exposure to GST-HG171 administered concomitantly with ritonavir progressively increased. To prevent excessive drug exposure, dose adjustments were necessary for individuals with mild or moderate renal dysfunction compared with those with normal renal function. Considering potential future tablet strengths of 50 mg and 150 mg, patients with mild renal impairment may receive 150 mg or 100 mg of GST-HG171 together with 100 mg of ritonavir, whereas patients with moderate renal impairment may be treated with 100 mg or 50 mg of GST-HG171 with 100 mg of ritonavir.

**Fig 2:**
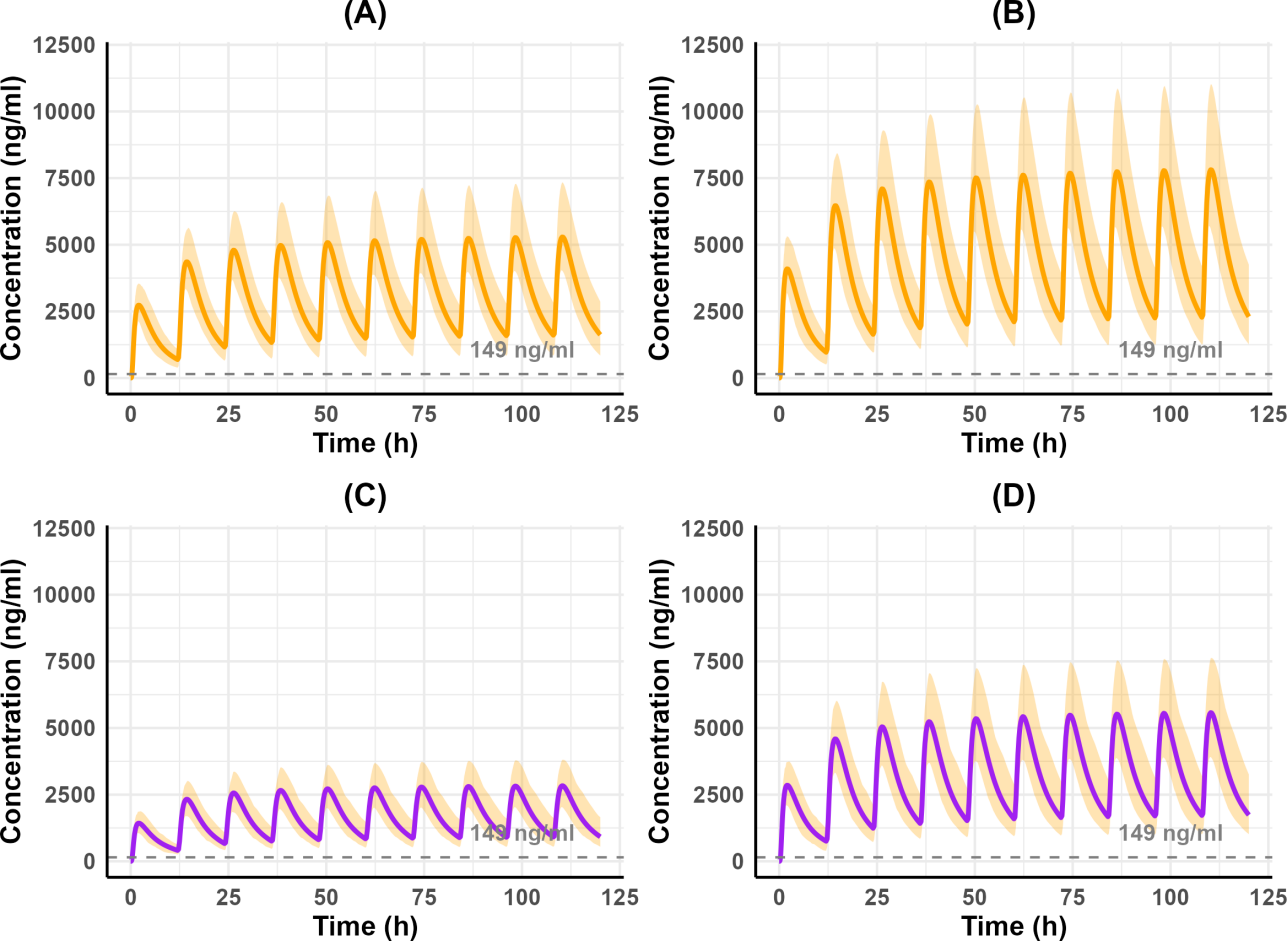
PBPK model simulations of GST-HG171/ritonavir in subjects with renal impairment. Simulated plasma concentrations of GST-HG171 in a virtual adult population (50% female) with mild or moderate renal impairment after 5 consecutive days of twice-daily co-administration with ritonavir. Solid lines represent mean concentrations, and shaded areas represent the 5th–95th percentile range. Simulation scenarios: (**A**) 100/100 mg in mild impairment, (**B**) 150/100 mg in mild impairment, (**C**) 50/100 mg in moderate impairment, and (**D**) 100/100 mg in moderate impairment.

Simulations of twice-daily dosing for 5 days showed that regimens of 100/100 mg GST-HG171/ritonavir for mild renal impairment and 50–100/100 mg for moderate renal impairment maintained trough concentrations above the effective concentration—the plasma protein–corrected EC_90_ value of 149 ng/mL—for GST-HG171, while remaining below the highest clinically observed steady-state *C*_max_ (9,547 ng/mL) identified in the Phase I clinical study (Fig. 2 and Table S3). These dosing strategies are expected to achieve therapeutic antiviral levels while minimizing the risk of excessive exposure—consistent with the recommended regimen in the prescribing information, except for a reduced dose of GST-HG171.

## DISCUSSION

GST-HG171 is an effective, orally administered inhibitor of the 3CL protease, approved in China for the treatment of mild to moderate COVID-19. CKD is known to reduce hepatic drug clearance by inhibiting cytochrome P450 enzymes (notably CYP3A4) and transporter activity, thereby altering systemic exposure. The higher GST-HG171 plasma levels observed in subjects with mild and moderate renal impairment are consistent with this mechanism (13, 14). The Fe_₀–₇₂h_ of approximately 40% indicates that urinary excretion serves as an important elimination route. However, these data do not confirm that renal excretion is the predominant pathway during co-administration with ritonavir. As presented in Table 2, the 0–72 h cumulative urinary excretion fractions of the parent drug were broadly comparable across the normal renal function, mild renal impairment, and moderate renal impairment groups. This observation indicates that glomerular filtration rate (GFR) exerts only a minimal effect on the renal clearance of GST-HG171.

Pharmacokinetic evaluation showed that *T*_max_ was unchanged across renal function groups, but elimination half-life (*t*_1/2_) was prolonged in subjects with renal impairment. Compared with normal renal function, mild renal impairment increased *C*_max_ by approximately 18% and AUC by approximately 38%–39%. Moderate renal impairment resulted in larger increases, with *C*_max_ up by approximately 31% and AUC by approximately 82%–86%. These changes were clinically meaningful and indicated reduced clearance of GST-HG171 in renal impairment (18).

Simulation results suggested that trough plasma concentrations of GST-HG171 in moderate renal impairment remained within the therapeutic index of 149–9,547 ng/mL for the dosing regimen of 50–100/100 mg (GST-HG171/ritonavir).

Given the available formulations of GST-HG171 (50 mg and 150 mg), only dosing regimens of 50 mg, 100 mg, and 150 mg are clinically feasible. The selected regimens were required to maintain steady-state GST-HG171 concentrations above the effective concentration and below the maximum observed steady-state safety concentration (149–9547 ng/mL). For patients with mild renal impairment, the 95th percentile of steady-state peak concentration following the 150/100 mg (GST-HG171/ritonavir) regimen reached the upper safety limit. Accordingly, the 100/100 mg regimen was deemed appropriate (Fig. 2 and Table S3). For patients with moderate renal impairment, both 50/100 mg and 100/100 mg regimens yielded drug concentrations within the target therapeutic window. Considering the renal status of this patient group, the 50/100 mg regimen was chosen to reduce the burden on impaired kidneys. Notably, the simulated exposure data exhibited high variability, introducing considerable uncertainty into the predictions. Further clinical studies in real-world patients with renal impairment will be conducted to optimize dosing strategies in this population.

The simulated geometric mean concentration with 50/100 mg dosing was comparable to the steady-state trough concentration achieved with the standard clinical regimen of 150/100 mg GST-HG171/ritonavir, averaging approximately 1,000 ng/mL. These results suggest that for COVID-19 patients with mild renal impairment, a recommended regimen could be 100 mg of GST-HG171 with 100 mg of ritonavir twice daily for 5 days, whereas those with moderate impairment may receive 50 mg of GST-HG171 with 100 mg of ritonavir twice daily. In comparison, the approved dosing adjustment for nirmatrelvir/ritonavir in moderate renal impairment is 150/100 mg twice daily for 5 days, a regimen that has demonstrated the efficacy and safety of Paxlovid (18, 19). Given the stronger *in vitro* antiviral activity of GST-HG171 compared with nirmatrelvir, a lower dose of GST-HG171 may be reasonable in patients with renal impairment (10).

The safety assessment indicated that GST-HG171 was generally tolerated in individuals with normal renal function and in those with mild-to-moderate renal impairment. However, adverse reactions related to hypertriglyceridemia require monitoring. Among the 24 subjects, 7 (29.2%) experienced 12 treatment-related TEAEs, most of which were mild. There were no serious events or discontinuities. Hypertriglyceridemia occurred in two participants with moderate impairment; all other adverse events were isolated cases. Headache, dry mouth, asthenia, and dysgeusia were infrequent; these events are often reported in patients with renal impairment after nirmatrelvir/ritonavir treatment (9, 20–22).

The incidence of adverse events (AE) in the moderate renal impairment group was slightly higher; however, most events were Common Terminology Criteria for Adverse Events (CTCAE) grade 1. Two subjects each experienced one episode of hypertriglyceridemia, which were CTCAE grade 2 and grade 3, respectively, and both had baseline triglyceride levels classified as grade 1. Subject B001 had a triglyceride level of 3.32 mmol/L (upper limit of normal [ULN] = 1.8 mmol/L) during the screening period, which was CTCAE grade 1. On Day 4 after administration, the level increased to 8.06 mmol/L, and on Day 5, it was 8.39 mmol/L, reaching CTCAE grade 3. Fenofibrate capsules 200 mg once daily were then administered orally from Day 5 to Day 10. By Day 10, the triglyceride level decreased to 1.85 mmol/L, and fenofibrate was discontinued. Subject B006 had a triglyceride level of 2.30 mmol/L (CTCAE grade 1) during the screening period. On Day 4 after administration, it increased to 4.29 mmol/L (CTCAE grade 2), and no lipid-lowering treatment was given. By Day 8, the level decreased to 1.84 mmol/L, returning to the normal range. The *C*_max_ and AUC_0_*_−t_* of B001 and B006 were 5,598.677 ng/mL and 6,253.444 ng/mL, 60,350.55 h·ng/mL and 79,913.31 h·ng/mL, respectively, both close to the group mean in the moderate renal impairment group. No clear exposure-dependent relationship was observed between drug exposure and hypertriglyceridemia.

The prescribing information for ritonavir indicates that lipid abnormalities (including hypercholesterolemia and hypertriglyceridemia) may occur after administration. In addition, renal function decline itself may contribute to disorders of lipid metabolism and elevated triglyceride levels. Given the limited sample size of this study, the causal relationship between GST-HG171 and elevated triglycerides remains unclear. Further evaluation in larger cohorts, including real-world patients with renal impairment, is warranted. Overall, GST-HG171/ritonavir was safe and tolerated in subjects with renal impairment.

The currently approved dosing schedule for GST-HG171 involves a 150 mg dose co-administered with 100 mg of ritonavir, given twice daily for 5 days. Although this study has limitations, including a small sample size and a single-dose design, it provides important pharmacokinetic data to support dose adjustments in patients with mild to moderate renal impairment. Post-marketing studies with repeated dosing in COVID-19 patients with mild to moderate renal impairment are warranted to further evaluate safety, with particular attention to laboratory parameters, including hematology, biochemistry, urinalysis, and renal function. This study did not measure ritonavir concentrations, and its dosing regimen was inconsistent with the commonly used twice-daily regimen in clinical practice, thereby failing to fully simulate the real clinical treatment scenario. In future real-world studies, the above pharmacokinetic information of patients with renal impairment should be included and combined with safety and efficacy data, to explore and establish an optimized dosing regimen suitable for these patients.

### Conclusion

This study showed that mild and moderate renal impairment significantly increased systemic exposure to GST-HG171 in Chinese subjects. These results support dose adjustment in clinical practice: 100/100 mg GST-HG171/ritonavir twice daily for patients with mild impairment and 50/100 mg twice daily for those with moderate impairment. GST-HG171 was generally tolerated after single-dose administration across all renal function groups. However, adverse reactions related to hypertriglyceridemia should be monitored.

## MATERIALS AND METHODS

### Study design

This clinical trial assessed the pharmacokinetic profile and safety of a single oral dose of GST-HG171 (150 mg) co-administered with ritonavir (100 mg) in individuals with mild to moderate renal dysfunction, compared with healthy subjects (ClinicalTrials.gov identifier: NCT06106126). The study used a nonrandomized, open-label, parallel-group, single-dose design and was conducted at a single center. The trial was carried out at the Phase I Clinical Research Center of the First Hospital of Jilin University (Jilin, China) from 22 December 2022 to 3 February 3, 2024.

A total of 24 participants were enrolled and assigned to 3 groups based on renal function: mild impairment (GFR 60–89 mL/min), moderate impairment (GFR 30–59 mL/min), and normal renal function (GFR > 90 mL/min), with 8 individuals in each group. Healthy controls were matched to the renal impairment groups by sex, body weight (within ±10 kg of the group mean), and age (within ±10 years of the group mean). Screening was performed within 14 days before drug administration. All participants were admitted to the Center on Day 1.

To achieve sufficient CYP3A inhibition before GST-HG171 administration, each participant received a single 100 mg oral dose of ritonavir on the evening of Day −1. Following an overnight fast of at least 10 h, participants received 150 mg GST-HG171 and 100 mg ritonavir on the morning of Day 1 (12 h after the initial ritonavir dose) with approximately 240 mL of warm water. To sustain CYP3A inhibition, additional 100 mg doses of ritonavir were administered at 12 and 24 h after GST-HG171 dosing. Subjects were required to abstain from drinking water for 1 h before and after receiving GST-HG171 and from eating for 2 h after drug administration. They remained seated for 2 h post-dose and limited physical activity to bed rest. Discharge occurred on Day 4, followed by a safety follow-up on Day 10 (±1 day) via telephone or text message.

Safety evaluations included physical examinations, monitoring of vital signs, laboratory analyses, 12-lead electrocardiograms, and continuous recording of adverse events throughout the study period. The study protocol and informed consent documents were reviewed and approved by the Ethics Committee of the First Hospital of Jilin University. The trial was conducted in compliance with the principles of the Declaration of Helsinki, Good Clinical Practice guidelines for pharmaceutical clinical trials, and all relevant regulations in China. Written informed consent was obtained from all subjects before any screening procedures.

### Subjects

Eligible participants were Chinese men or women aged 18 to 70 years with a body mass index (BMI) ranging from 18 to 32 kg/m². All individuals agreed to employ effective contraceptive methods starting from the screening phase and continuing for up to 6 months after the last dose. Inclusion criteria were as follows: (i) healthy controls: demonstrated normal physical examination results, stable vital signs, and electrocardiogram (ECG) findings or exhibited minor abnormalities deemed not clinically relevant and had a glomerular filtration rate (GFR) exceeding 90 mL/min. (ii) Renal impairment subjects: individuals with chronic kidney disease and stable renal function, confirmed by two GFR tests within the same CKD stage, performed at least 3 days apart before dosing. Individuals with mild renal impairment had a GFR of 60–89 mL/min, whereas those with moderate impairment had a GFR of 30–59 mL/min. Aside from renal dysfunction and associated complications, individuals were considered to be in stable general health based on review of medical history, physical examination, laboratory test results, and electrocardiogram findings.

Exclusion criteria included: (i) use of strong or moderate CYP3A, or P-glycoprotein inducers or inhibitors, within 30 days before screening; (ii) pregnant or lactating women, or females of reproductive potential who tested positive on a pregnancy test; (iii) active infection with hepatitis B virus or hepatitis C virus, infection with HIV, or a positive *Treponema pallidum* antibody test; and (iv) additional exclusion criteria specific to individuals with renal impairment: acute renal failure, history of kidney transplantation, anticipated need for dialysis during the study period, alanine aminotransferase or aspartate aminotransferase levels more than 2× the upper limit of normal (ULN), total serum bilirubin more than 1.5× ULN, albumin less than 30 g/L, hemoglobin less than 60 g/L, heart failure with New York Heart Association functional class III or IV, or left ventricular ejection fraction less than 50% at screening.

### Pharmacokinetic sampling and analysis

For pharmacokinetic (PK) assessment, venous blood samples (4 mL each) were collected into vacuum tubes containing EDTA-K₂ anticoagulant at the following time points: Day 1 (0 h, predose) and 0.25, 0.5, 0.75, 1, 1.25, 1.5, 2, 3, 4, 6, 8, 12, 24, 48, and 72 h after administration. The blood samples were centrifuged at 1,500*g* for 10 min at 2°C–8°C to obtain plasma. Urine samples were collected at baseline (0 h, before administration on Day 1) and at 0–6 h, 6–12 h, 12–24 h, 24–48 h, and 48–72 h after administration.

Plasma concentrations of GST-HG171 were measured using a validated liquid chromatography–tandem mass spectrometry method with adequate sensitivity and specificity. Method validation was conducted by Frontage Laboratories (Suzhou) Co., Ltd., and the validation report is on file. The calibration curve ranged from 10 to 10,000 ng/mL, with a lower limit of quantification of 10 ng/mL. The bioanalytical method demonstrated acceptable precision and accuracy, meeting regulatory requirements for assay validation. Pharmacokinetic analysis was conducted with Phoenix WinNonlin (version 8.3), using noncompartmental analysis to estimate (PK) parameters based on the actual recorded sampling times.

PK parameters included maximum observed plasma concentration (*C*_max_), terminal elimination half-life in plasma (*t*_1/2_), time to maximum observed plasma concentration (*T*_max_), area under the concentration–time curve (AUC) from time of dosing (0 h) to time *t* h (AUC_0–*t*_), or from time of dosing extrapolated to infinity (AUC_0–∞_), apparent clearance (CL/F), and renal clearance (CLr).

### Safety assessments

Safety assessments were conducted by monitoring adverse events (AEs), performing physical examinations, measuring vital signs, conducting laboratory evaluations, and obtaining 12-lead ECG. For each reported AE, detailed information was documented, including severity, onset and resolution times, duration, interventions, and outcome, along with an evaluation of the potential relationship to the study drug. The intensity of AEs was graded according to the CTCAE version 5.0. Treatment-emergent adverse events were monitored throughout the 4-day inpatient period at the clinical research unit and followed up by a telephone interview on Day 10 (±1 day).

### Development of the PBPK model

A PBPK model for GST-HG171 was constructed, validated, and used for dose-simulation studies with the open-source software PK-Sim (version 12.0, part of the Open-Systems-Pharmacology suite). Data visualization and graphics were performed in R (version 4.4.2, R Foundation for Statistical Computing, Vienna, Austria) using RStudio (version 2023.09.1-494, Posit, Inc., Boston, USA) (23, 24). Comprehensive methodological details are provided in the Supplementary Materials. Final PBPK model parameters for GST-HG171 are summarized in Table S1.

### Statistical analysis

Descriptive statistics were used to summarize demographic, safety, and PK data. Continuous variables are expressed as mean ± standard deviation (SD), and categorical variables are reported as frequency counts with corresponding percentages. To compare PK parameters (*C*_max_, AUC_0–*t*_, and AUC_0–∞_) across renal function groups, a mixed-effects analysis of variance (ANOVA) model was applied to log-transformed data; least-squares means, and 90% confidence intervals (CIs) were estimated accordingly. Safety outcomes were summarized descriptively based on event incidence, severity, and the assessed relationship to the investigational drug. All statistical analyses were conducted using SAS software (version 9.4; SAS Institute Inc., Cary, NC, USA).

## SUPPLEMENTAL MATERIAL

Supplemental material aac.01541-25-s0001.docx Tables S1 to S3; Fig. S1.

ASM does not own the copyrights to Supplemental Material that may be linked to, or accessed through, an article. The authors have granted ASM a non-exclusive, world-wide license to publish the Supplemental Material files. Please contact the corresponding author directly for reuse.
