# Supplementary material for: Pharmacokinetics, safety, and dose optimization of GST-HG171 in renal impairment: insights from physiologically based pharmacokinetic modeling
Source: Antimicrob Agents Chemother. 2026 Jul 15;70(8):e01541-25. doi: 10.1128/aac.01541-25 (PMC13435689; doi:10.1128/aac.01541-25)
Supplement: Supplementary file 1 [file aac.01541-25-s0001.docx]

# Supplement materials

## Development and verification of the PBPK model

The ritonavir model was adapted from Liang Zheng et al.^1^, who established a PBPK model that accounts for CYP3A4 induction, competitive and time-dependent inhibition, as well as competitive inhibition of CYP2D6. Parameters related to oral administration, specifically the “dissolution time (50%)” and “dissolution shape,” were optimized to 60 min and 0.71, respectively. The predicted values of Cmax and AUC for ritonavir were consistent with observed values, at 2227 vs. 2000 ng/mL for Cmax and 14,684 vs. 18,700 ng·h/mL for AUC^2^.

The PBPK model of GST-HG171 was first validated using PK data from single-dose administration of GST-HG171 tablets and from co-administration of GST-HG171 with ritonavir in healthy subjects^3^. The model was further verified against PK parameters obtained from co-administration of GST-HG171 and ritonavir in subjects with mild renal impairment (GFR 60–89 mL/min) and moderate renal impairment (GFR 30–59 mL/min), estimated using the CKD Epidemiology Collaboration (CKD-EPI) equation.

Ratios of predicted-to-observed values of Cmax and AUC were used to assess the model’s fit against individual clinical trial results. Sensitivity analyses and parameter optimization were performed to refine model accuracy. Model performance was quantitatively assessed using the GMFE for AUC and Cmax values (Equations 1–2). A GMFE within a twofold range was predefined as indicating successful model prediction^2^.

$GMFE={10}^{\left( \sum\left| \log_{10} ratio \right| \right)/n}$	(Equation 1)

$ratio={{PARM}_{pred}}/{{PARM}_{obs}}$	(Equation 2)

with n=number of studies, PARM_pred=_predicted AUC and C_max_ value, PARM_obs_=corresponding observed AUC and C_max_ value

## Prospective simulations

Using the final modified PBPK model, prospective simulations were performed to predict plasma concentrations of GST-HG171 under different dosing regimens in patients with mild or moderate renal impairment (age distribution similar to the present study, with 50% female). The BID regimens simulated over 5 days were:

## Mild impairment: 150/100 mg or 100/100 mg (GST-HG171/ritonavir)

## Moderate impairment: 100/100 mg or 50/100 mg (GST-HG171/ritonavir)

The effective concentration (EC90) of GST-HG171 against SARS-CoV-2 is 149 ng/mL, while the maximum tolerated plasma Cmax confirmed in Phase I trials is 9547 ng/mL. Therefore, plasma trough concentrations between 149 and 9547 ng/mL are required to maintain therapeutic activity^3^. For each simulated regimen, the 5th–95th percentile range of the concentration–time curve was evaluated to ensure that plasma levels remained within this predefined therapeutic window.

Table S1. Final PBPK model parameters for GST-HG171.

| Parameter | Value^a^ |
| --- | --- |
| Physiochemical properties | |
| Lipophilicity | 0.24 ^b^ |
| Molecular Weight (g/mol) | 511.55 |
| Halogens | no |
| Fraction Unbound | 0.27 |
| Protein Binding | Albumin |
| Solubility (mg/l) | 639.48 ^b^ |
| pKa | neutral |
| Specific intestinal perm (cm/min) | 1.25E-05 |
| Partition coefficients | Rodgers and Rowland |
| Cellular permeabilities | PK-Sim Standard |
| Specific organ perm | PK-Sim Calculated |
| Formulation | Weibull |
| Dissolution time (min) | 72.94 ^b^ |
| Dissolution shape | 0.84^b^ |
| Metabolism and Transport | |
| CYP2C8 specific clearance (1/mn) | 0.03 |
| CYP2C9 specific clearance (1/mn) | 0.03 |
| CYP2D6 specific clearance (1/mn) | 3.23E-03 ^b^ |
| CYP3A4 specific clearance (1/mn) | 14.35(healthy subject) ^b^;2.86(renal impairment subject)^b^ |
| GFR | 1^c^ |

^a^GST-HG171 unannotated parameters are unpublished preclinical data provided by Fujian Akeylink Biotechnology Co., Ltd., Fuzhou, Fujian, China; ^b^Optimized value; ^c^Assumed value;

Table S2. Observed and simulated pharmacokinetic parameters of GST-HG171/ritonavir in healthy subjects and subjects with renal impairment.

| Subject | Dose (mg) | AUC_0-24_ (ng/mL*h) | | | C_max_ (ng/mL) | | |
| --- | --- | --- | --- | --- | --- | --- | --- |
|  |  | Predicted | Observed | Ratio | Predicted | Observed | Ratio |
| healthy subject | 150, SAD^a^ | 5960.475 | 5676.96 | 1.05 | 1467.538 | 1128.63 | 1.3 |
| healthy subject | 300, SAD^a^ | 11916.15 | 11890.92 | 1 | 2935.035 | 3018.97 | 0.97 |
| healthy subject | 150, DDI, day 1 ^b^ | 20332.81 | 22766.44 | 0.89 | 3683.822 | 3354.14 | 1.1 |
| healthy subject | 150, DDI, day 5^b^ | 41108.54 | 30641.19 | 1.34 | 6590.552 | 4387.91 | 1.5 |
| healthy subject | 150, DDI, day 1^c^ | 29691 | 35311.94 | 0.84 | 4638.548 | 3790.94 | 1.22 |
| mild renal impairment subject | 150, DDI, day 1 ^c^ | 48715.56 | 48275.44 | 1.01 | 5675.508 | 4568.3 | 1.24 |
| moderate renal impairment subject | 150, DDI, day 1 ^c^ | 51300.61 | 61111.49 | 0.84 | 5930.637 | 5452.21 | 1.09 |

^a:^ GST-HG171 150 or 300 mg single dose at 0 h^3^;

^b:^ 5 days of BID administration regimen of 150/100 mg GST-HG171/ritonavir^3^;

^c^: GST-HG171 150 mg single dose at 12 h and 4 doses of ritonavir 100 mg 0, 12, 24, 36 h;

Table S3. Simulated pharmacokinetic parameters of GST-HG171/ritonavir administered twice daily at steady state on day 5 in healthy subjects and subjects with renal impairment

| Group | Cmax (ng/mL) | AUC_0-12_ (ng/mL*h) | Cmin (ng/mL) |
| --- | --- | --- | --- |
| A | 5290 (189%) | 17348 (582%) | 1608 (376%) |
| B | 7813 (207%) | 58050 (274%) | 2279 (393%) |
| C | 2834 (191%) | 21736 (260%) | 911 (363%) |
| D | 5576 (203%) | 42086 (279%) | 1721 (389%) |

CV% = (Standard Deviation / Mean) × 100%; Data are presented as mean (CV%)

A: GST-HG171 100 mg / ritonavir 100 mg in subjects with mild renal impairment

B: GST-HG171 150 mg / ritonavir 100 mg in subjects with mild renal impairment

C: GST-HG171 50 mg / ritonavir 100 mg in subjects with moderate renal impairment

D: GST-HG171 100 mg / ritonavir 100 mg in subjects with moderate renal impairment

Figure S1. Simulation results of the GST-HG171/ritonavir PBPK model in Phase I healthy subjects^3^ and in subjects with renal impairment.

Solid lines represent simulated mean plasma concentrations; shaded areas represent the 5th–95th percentile range.

•	Panel A: GST-HG171 150 mg single dose in healthy subjects (0 h)

•	Panel B: GST-HG171 300 mg single dose in healthy subjects (0 h)

•	Panel C: GST-HG171/ritonavir 150/100 mg BID for 5 days in healthy subjects

•	Panel D: GST-HG171/ritonavir in subjects with normal renal function

•	Panel E: GST-HG171/ritonavir in subjects with mild renal impairment (eGFR 60 to <90 mL/min)

•	Panel F: GST-HG171/ritonavir in subjects with moderate renal impairment (eGFR 30 to <60 mL/min)

references

[1]	Zheng L, Zhang W, Olkkola KT, et al. Physiologically based pharmacokinetic modeling of ritonavir-oxycodone drug interactions and its implication for dosing strategy. Eur J Pharm Sci. 2024. 194: 106697.

[2]	Sun Z, Zhao N, Xie R, et al. Physiologically-based pharmacokinetic modeling predicts the drug interaction potential of GLS4 in coadministered with ritonavir. CPT Pharmacometrics Syst Pharmacol. 2024. 13(9): 1503-1512.

[3]	Zhang H, Zhou J, Chen H, et al. Phase I study, and dosing regimen selection for a pivotal COVID-19 trial of GST-HG171. Antimicrob Agents Chemother. 2024. 68(1): e0111523.
